# Prehospital Injury Severity Estimate (PHISE) matches in-hospital trauma scores when embedded in AI models

**DOI:** 10.1038/s41746-026-03074-7

**Published:** 2026-08-07

**Authors:** Manuel Sigle, Andreas Goldschmied, Meinrad Gawaz, Alexander Münch, Peter Rosenberger, preTransIT Investigator group, Sven Weisser, Andreas Gather, Alfred Grützner, Oliver Kamp, Christian Waydhas, Marcel Dudda, André Nohl, Nicolas Eibinger, Paul Puchwein, Saffet Özkaya, Christian Knop, Martin Breitkopf, Kai Oliver Jensen, Yannik Kalbas, Ferdinand C. Wagner, Benjamin Erdle, Robert Wunderlich

**Affiliations:** 1 Department of Cardiology and Angiology, University Hospital Tübingen, Eberhard Karls University Tübingen, Tübingen, Germany; 2 University Department of Anesthesiology and Intensive Care Medicine, University Hospital Tübingen, Eberhard Karls University Tübingen, Tübingen, Germany; 3 Department for Paraplegics, Technical Orthopedics and Spinal Surgery, BG Tübingen Hospital, Tübingen, Germany; 4 BG Ludwigshafen Hospital, Ludwigshafen, Germany; 5 Department of Trauma, Hand and Reconstructive Surgery, University Hospital, Essen, Germany; 6 Department of Orthopedics and Trauma Surgery, BG Klinikum Duisburg, University of Duisburg-Essen, Essen, Germany; 7 Zentrum für Notfallmedizin, BG Klinikum Duisburg, Duisburg, Germany; 8 Department of Orthopedics and Trauma, Medical University of Graz, Graz, Austria; 9 Department of Trauma Surgery and Orthopedics, Klinikum Stuttgart-Katharinenhospital, Stuttgart, Germany; 10 Department of Anesthesiology, Operative Intensive Care Medicine, Emergency Medicine and Pain Medicine, Klinikum Stuttgart-Katharinenhospital, Stuttgart, Germany; 11 Trauma Department Zürich, University Hospital Zürich, Zürich, Switzerland; 12 Hospital Bülach AG, Bülach, Switzerland; 13 Department of Orthopedics and Traumatology, Freiburg University Hospital, Freiburg, Germany

**Keywords:** Diseases, Health care, Medical research

## Abstract

Trauma severity scores such as the Injury Severity Score (ISS) and New Injury Severity Score (NISS) are widely used for trauma benchmarking, risk stratification and outcome prediction, but rely on diagnostic imaging unavailable in the prehospital setting. We developed and externally validated the Prehospital Injury Severity Estimate (PHISE), a simple, scene-based score designed to be applied using clinical examination and knowledge of the injury mechanism. PHISE was retrospectively derived from the National Trauma Data Bank® (NTDB®) by mapping AIS-coded injury descriptions to eight PHISE body regions and four severity levels, using Large Language Model-assisted inference to identify imaging requirements and distinguish clinically assessable injuries. In the NTDB® cohort, PHISE score demonstrated strong correlation and calibration with ISS and NISS but lower predictive performance for outcomes including in-hospital mortality and blood transfusion need. External validation used the multinational TraumaRegister DGU®, which reports real-world data of a simplified prehospital injury score compatible to PHISE. Here, PHISE underperformed ISS/NISS in predicting mortality but achieved comparable performance for transfusion prediction. When embedded into machine learning models alongside prehospitally available patient variables, the performance gap to ISS/NISS narrowed further. PHISE provides a pragmatic, imaging-independent anatomical component for earlier AI-assisted trauma stratification in prehospital emergency care.

## Introduction

Trauma remains a leading cause of death and disability worldwide, requiring rapid and accurate assessment of injury severity to guide triage, resource allocation, and treatment decisions. The Injury Severity Score (ISS)^1^ and its derivative, the New ISS (NISS)^2^, have long served as reference standards for anatomical injury quantification in trauma care, benchmarking and research. Early efforts to facilitate clinical applicability included condensed AIS charts enabling more rapid bedside injury scaling and ISS calculation shortly after admission^3^. However, both ISS and NISS ultimately rely on detailed anatomical coding and typically require advanced imaging—particularly computed tomography (CT)—making them impractical in the early, prehospital phase of care^4–6^.

The absence of a validated, standardized method for estimating injury severity in the prehospital setting represents a critical gap in trauma systems worldwide. Field providers are often forced to rely on non-standardized clinical gestalt and patient examination to estimate injury severity, which may miss the nuance and complexity of real injury burden. This limits the ability to make time-critical decisions such as trauma team activation, hospital bypass, or resource mobilization.

We hypothesized that a simplified, region-based injury severity score could approximate ISS/NISS performance when applied to solely clinically accessible information at the scene. To address this, we developed the Prehospital Injury Severity Estimate (PHISE), a score based on eight anatomically distinct body regions and a four-level injury grading scale (no/minor/moderate/severe injury). PHISE is intended to complement physiological assessment and clinical judgment, not to replace them.

We evaluated PHISE in two large trauma datasets – retrospectively for the US-based National Trauma Data Bank® (NTDB®) and prospectively for the German-speaking countries’ TraumaRegister DGU® – assessing its correlation, calibration, and predictive performance relative to ISS and NISS. We further embedded PHISE into machine learning models alongside vital signs and injury descriptors to explore its added value in contemporary, AI-assisted decision-support frameworks.

This study demonstrates the feasibility of a pragmatic, radiology-imaging-independent severity score that can be captured at the scene and evaluated for integration into digital decision-support tools for emergency and critical care medicine.

## Results

### Injury characteristics and imaging Requirements

**Fig. 1:**
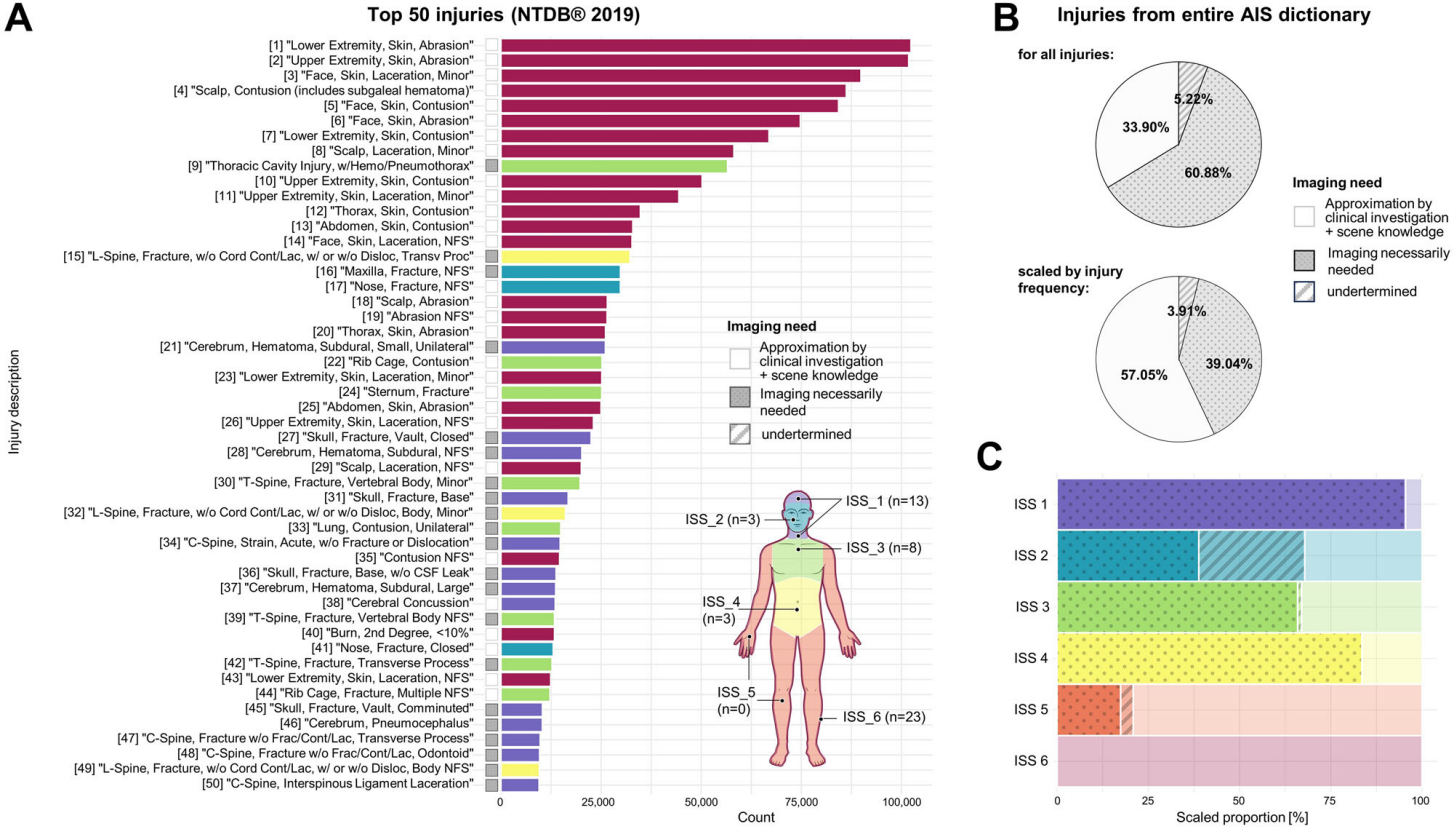
Prevalence and imaging need of top injuries in the NTDB® dataset. **A** Distribution of the 50 most frequent injury codes in the NTDB® 2019 dataset, categorized by body region (bar color). An anatomical reference map displays the mapping of AIS-defined body regions per color to the six ISS body regions. The texture indicator refers to the estimated imaging need, as identified using a Large Language Model. **B** Pie charts show the proportion of injuries that can be approximated by scene investigation, those requiring diagnostic imaging, and those marked as undetermined. The top pie reflects all AIS dictionary entries, the bottom one weights injuries by their real-world frequency in the NTDB® 2019. **C** Stacked bar chart showing the scaled proportion of AIS severity grades for each ISS body region (ISS 1–6). Fill patterns indicate imaging need.

The 2019 National Trauma Data Bank (NTDB®) dataset comprised 397,864 trauma patients with complete AIS-based injury coding and ISS/NISS scores. For descriptive characterization of injury patterns, we identified the 50 most frequent injury codes and grouped them by ISS body region and clinical ability to assess. A large majority of these injuries occurred in body regions accessible by physical examination, including the extremities, head, and thorax. To estimate imaging dependency, we used Large Language Model (LLM)-assisted inference on AIS-coded injury descriptions. Injuries were categorized as: (1) assessable by scene and clinical knowledge, (2) requiring diagnostic imaging, or (3) undetermined. LLM classification revealed that, after scaling by injury frequency, 57% of injuries could be identified without imaging, while 39% relied on imaging, and 4% remained ambiguous (Fig. 1A, B). Categorized by body region, injuries in ISS6 (external) did require nearly no imaging. In contrast, head and abdominal/pelvic injuries demonstrated the highest proportion of injuries requiring imaging (Fig. 1C). The fact that most of the top 50 injuries covered body regions and injury patterns that were also assessable by clinical investigation and scene knowledge, this supports the feasibility of scene-based injury scoring.

### Cohort derivation and structural comparison of PHISE and ISS

**Fig. 2:**
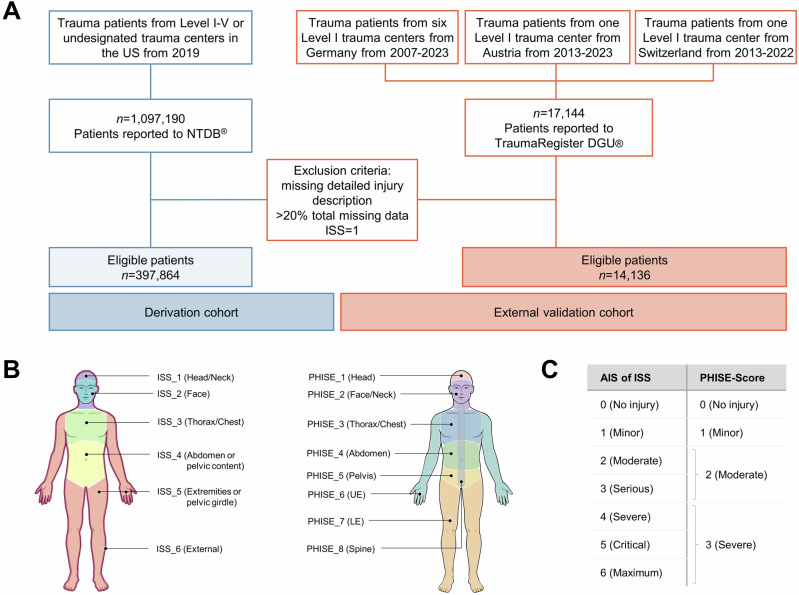
Derivation of the study population and comparison of ISS and PHISE structure. **A** Flowchart illustrating patient selection for the derivation and validation cohorts. Inclusion was based on the availability of detailed injury descriptions and prehospital variables. **B** Comparison of anatomical regions covered by the traditional ISS (left) and the proposed PHISE (right). PHISE captures eight body regions, which are distinctly identifiable through prehospital assessment. **C** Injury severity grading schemes for ISS (based on the Abbreviated Injury Scale, AIS) and PHISE. While AIS distinguishes six levels of severity (0–6), PHISE employs a simplified four-level scale (0–3) to improve prehospital applicability.

We included 1,097,190 trauma patients from the NTDB® 2019 dataset who had complete AIS-coded injury descriptions and valid ISS/NISS scores. After excluding patients with unmappable AIS entries or missing key prehospital variables, 397,864 patients remained for derivation of the PHISE score. The external validation cohort comprised 14,136 patients from the multinational TraumaRegister DGU®, which features a structured prehospital documentation^4^ compatible with PHISE logic (Fig. 2A).

Among derivation cohort patients, 21.8% had severe trauma (ISS > 15), compared to 54.4% in the validation cohort (Table 1). Median patient age was slightly lower in the NTDB® (50 years [IQR 26–69]) than in the TraumaRegister DGU® (51 years [IQR 31–67]), while the proportion of female patients was lower in the validation cohort (28.6% vs. 37.4%). Injury patterns revealed higher median ISS and NISS scores in the validation cohort, especially in thoracic and extremity injuries. Median PHISE scores also increased accordingly (9 [IQR 5–14] vs. 13 [IQR 9–22] in ISS > 15 subgroups). Across body regions, the NTDB® cohort exhibited slightly higher values. Clinical outcomes differed substantially: mortality was 4.4% in the NTDB® versus 13.0% in the TraumaRegister DGU®, and transfusion rates were 6.9% vs. 10.7%, respectively, reflecting a greater injury burden and preselection of severely injured patients in the European cohort (Table 1).

**Table 1:** Baseline characteristics of the derivation validation cohorts

	NTDB^®^ derivation cohort		TraumaRegister DGU® validation cohort	
	All patients	ISS > 15	All patients	ISS > 15
	*N* = 397 864	*N* = 86 561 (21.8%)	*N* = 14 136	*N* = 7 692 (54.4%)
Age	50 (29–69)	48 (29–66)	51 (31–67)	53 (35–70)
Female sex	148647 (37.4%)	26312 (30.4%)	4037 (28.6%)	2095 (27.2%)
Injury pattern				
ISS_1 (Head, Neck)	3 (3.06)	4 (3.75)	2 (1.91)	3 (2.69)
ISS_2 (Face)	1 (1.36)	1 (1.48)	0 (0.43)	0 (0.58)
ISS_3 (Thorax, Chest)	3 (2.46)	3 (2.85)	0 (1.45)	3 (2.07)
ISS_4 (Abdomen, pelvic content)	2 (2.20)	2 (2.51)	0 (0.62)	0 (0.87)
ISS_5 (Extremities, pelvic girdle)	2 (1.80)	2 (2.08)	1 (1.32)	2 (1.52)
ISS_6 (External)	1 (1.03)	1 (1.07)	0 (0.65)	0 (0.70)
PHISE_1 (Head)	1 (1.33)	1 (1.53)	1 (1.43)	2 (1.76)
PHISE_2 (Face/Neck)	1 (1.12)	1 (1.18)	0 (0.50)	0 (0.57)
PHISE_3 (Thorax/Chest)	1 (1.55)	1 (1.70)	0 (0.82)	0 (1.04)
PHISE_4 (Abdomen)	1 (1.49)	1 (1.65)	0 (0.34)	0 (0.43)
PHISE_5 (Pelvis)	2 (2.00)	2 (1.98)	0 (0.40)	0 (0.49)
PHISE_6 (UE)	1 (1.05)	1 (1.06)	0 (0.61)	0 (0.63)
PHISE_7 (LE)	1 (1.06)	1 (1.07)	0 (0.70)	0 (0.68)
PHISE_8 (Spine)	2 (2.00)	2 (2.00)	0 (0.54)	0 (0.60)
ISS	9 (5–14)	22 (17–27)	17 (9–25)	25 (19–33)
NISS	4 (2–14)	24 (16–34)	22 (12–34)	33 (24–43)
PHISE	4 (1–9)	9 (5–14)	10 (8–18)	13 (9–22)
Vital signs				
Systolic blood pressure	138 (120–157)	132 (110–153)	133 (114–150)	130 (110–150)
Respiratory rate (min^−1^)	18 (16–20)	18 (16–21)	15.00 (12–18)	15 (12–18)
GCS	15 (14–15)	14 (9–15)	15 (14–15)	14 (3–15)
Outcome				
Mortality (Death)	14 193 (4.4%)	10 988 (14.2%)	1835 (13.0%)	1657 (21.5%)
RBCC transfused	24 572 (6.9%)	17 724 (22.1%)	1512 (10.7%)	1305 (17.0%)

Continuous variables are given as median and interquartile range (IQR). For injury severities per body region, median with mean in brackets is displayed. Other ordinal variables including ISS, NISS, RTS and GCS are shown as median with IQR. Categorical variables are displayed as absolute and relative frequency within each transfusion category.

*RBCC* red blood cell concentrate, *ISS* Injury Severity Scale, *NISS* New Injury Severity Scale, *RTS* Revised Trauma Score, *GCS* Glasgow Coma Scale, *UE* Upper extremity, *LE* lower extremity.

PHISE differs structurally from ISS by expanding the number of anatomical regions from six to eight and by focusing on body regions that are more distinctly accessible through prehospital clinical examination. Compared to the traditional ISS grouping, PHISE separates the upper and lower extremities and adds the spine as an explicit region. The ISS category “external” was omitted, as these injuries are already captured within other anatomical regions and this structure better reflects the practical assessment processes used during primary and secondary surveys in the prehospital setting (Fig. 2B). In terms of severity grading, ISS relies on the Abbreviated Injury Scale (AIS), which assigns scores from 1 (minor) to 6 (unsurvivable), while PHISE uses a simplified four-level system ranging from 0 (no injury) to 3 (severe injury). This coarser grading scheme was intentionally designed to enhance practicality and consistency in time-critical prehospital environments (Fig. 2C).

### Retrospective derivation of PHISE and LLM-based imaging-need labeling

**Fig. 3:**
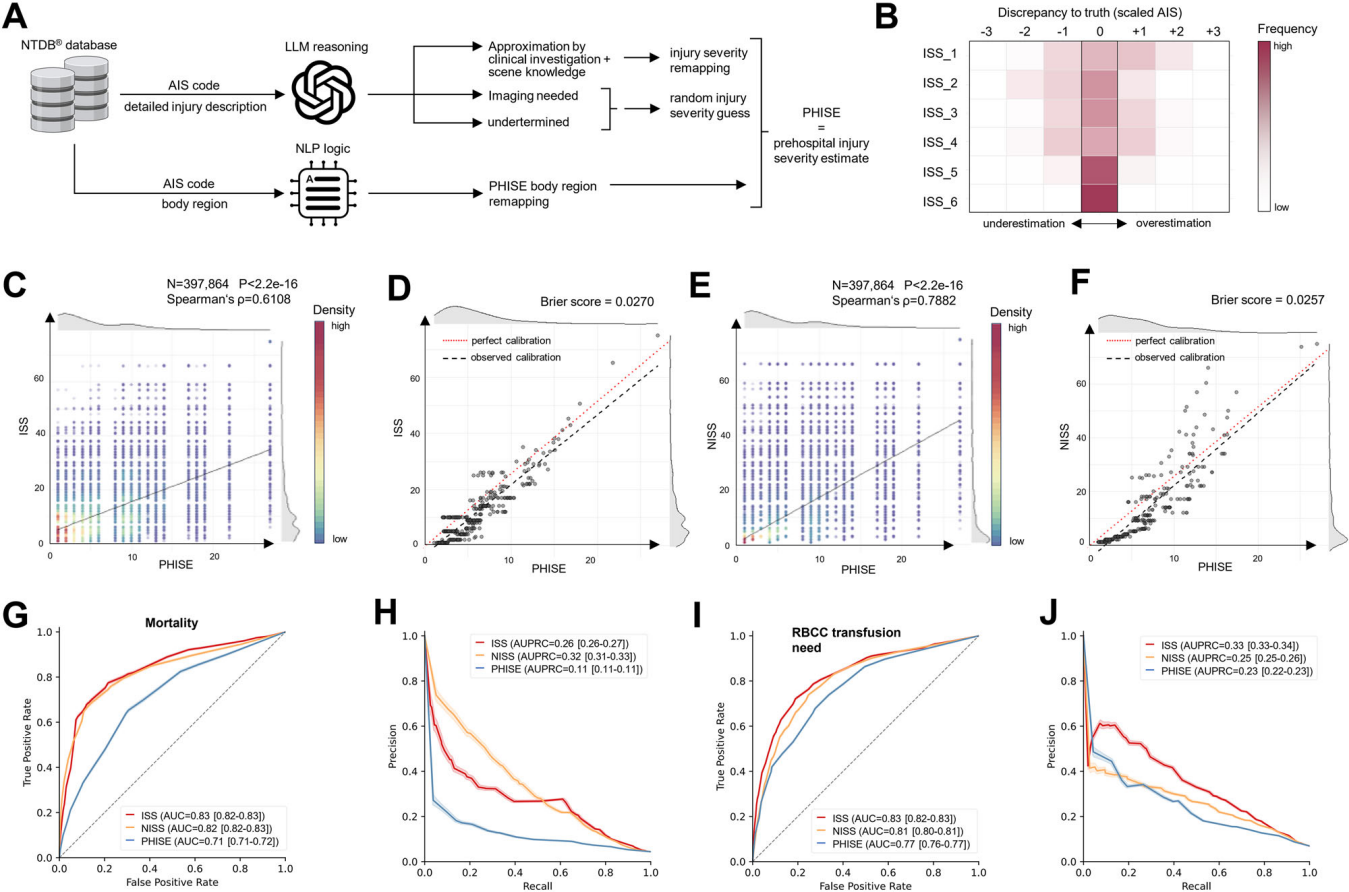
Estimating injury severity without imaging: PHISE calibration, correlation, and outcome prediction. **A** Schematic illustration of the logic used to derive PHISE scores from detailed AIS-coded injury descriptions of the NTDB® 2019. Large Language Model (LLM) reasoning and NLP-based remapping were used to infer imaging need and translate AIS body regions into PHISE body regions. Injuries requiring imaging or with undetermined imaging status were assigned randomly guessed PHISE severities, injuries assessable through clinical investigation or scene knowledge were reassigned to PHISE severity grades (0–3). **B** Region-wise discrepancy between rescaled AIS severities and mapped PHISE grades across all ISS body regions. ISS region 6 (external injuries) showed no discrepancy, as all relevant injuries can be identified without imaging. **C–F** Correlation and calibration of PHISE scores against traditional scores. Scatter plots show correlation of PHISE with ISS (**C**) and NISS (**E**). Panels **D** and F depict corresponding calibration plots using 1000-patient bins; the dashed line indicates ideal calibration. **G**, **H** Receiver operating characteristics and precision-recall analyses for ISS, NISS, and PHISE in predicting in-hospital mortality. **I**, **J** Receiver operating characteristics and precision-recall analyses for ISS, NISS, and PHISE in predicting red blood cell concentrate (RBCC) transfusion need. Shaded areas represent 95% confidence intervals.

To derive the Prehospital Injury Severity Estimate (PHISE) retrospectively, detailed injury descriptions from AIS codes we embedded into a standardized prompt (Supplementary Fig. S1) for ChatGPT (OpenAI model GPT-4o-mini, April 2025) questioning the necessity of imaging to allow the diagnosis as provided by the injury description (Fig. 3A). The large language model (LLM) then assigned one of three categories: (1) approximation by clinical investigation and scene knowledge, (2) imaging required and (3) undetermined. To assess output stability, the prompt was executed three times on the identical input dataset; when classifications differed, the final label was determined by majority vote. Model outputs were additionally reviewed by manual spot-checking for face validity.

Injuries classified as requiring imaging or marked as undetermined were randomly assigned a severity grade to simulate prehospital uncertainty. In contrast, injuries deemed clinically assessable were remapped from the original 6-level AIS scale to the simplified 4-level PHISE scale (see also Fig. 2C): AIS 0 was mapped to PHISE 0 (no injury); AIS 1 to PHISE 1 (minor); AIS 2-3 to PHISE 2 (moderate); and AIS 4–6 to PHISE 3 (severe). This grouping was chosen to maintain clinical interpretability while reducing granularity to a level compatible with prehospital assessment, where discrimination between higher AIS severities (4–6) is generally not feasible without imaging. To accommodate PHISE’s expanded anatomical framework of eight body regions, we implemented a natural language processing (NLP) logic to map AIS-coded body regions to the PHISE system. This approach enabled a fully automated, rule-based derivation of PHISE scores, supporting downstream validation and hypothesis testing.

### Concordance and calibration with ISS/NISS

To evaluate the accuracy of injury severity estimation without radiological imaging, we assessed the discrepancy between the derived PHISE grades and the ground truth AIS severity levels (rescaled to the four-level PHISE scale; Fig. 3B). As expected, the lowest discrepancy was observed in ISS region 6 (external), where superficial injuries are easily identifiable through clinical inspection. The highest discrepancy occurred in ISS region 4 (abdomen and pelvic contents) and 1 head/neck) reflecting the challenge of detecting intra-abdominal and intracerebral injuries that typically require cross-sectional imaging, such as CT scans. Surprisingly, PHISE showed good agreement in ISS region 3 (thorax), likely due to the lower variety of injury types in this region and the high visibility of certain conditions (e.g., pneumothorax), which can be detected through clinical examination or, where available, prehospital ultrasound.

We next compared the composite PHISE score – calculated as the squared sum of the three most severely affected body regions – to the established ISS and NISS scores. PHISE score showed good monotonic correlation with both ISS (Spearman’s ρ = 0.61; Fig. 3C) and NISS (ρ = 0.79; Fig. 3E). Calibration analyses using patient-wise binning in groups of 1,000 confirmed that PHISE score was reasonably aligned with ISS and NISS at the population level, although there was a tendency to overestimate lower-severity injuries (Fig. 3D, F). This overestimation likely reflects the coarser resolution of PHISE score (maximum score 27) compared to ISS/NISS (maximum score 75), as well as its reliance on clinically observable injuries.

Despite these structural limitations, PHISE calibration performance was acceptable, with Brier scores of 0.027 (vs. ISS) and 0.026 (vs. NISS), suggesting substantial agreement across the severity spectrum.

To assess temporal stability, we repeated all analyses in the 2020 NTDB® dataset, representing trauma care during the COVID-19 pandemic (Supplementary Figs. S2–S4). PHISE demonstrated comparable correlation, calibration, and predictive performance, indicating robustness even under pandemic-related system stress.

### Predictive performance in the derivation cohort

We next evaluated the ability of PHISE to predict clinically relevant outcomes, benchmarking its performance against the established ISS and NISS scores. For in-hospital mortality, PHISE demonstrated reasonable discriminative power (AUC = 0.71), although this was lower than that of ISS (AUC = 0.83) and NISS (AUC = 0.82; Fig. 3G). Precision-recall analysis (Fig. 3H) further emphasized this gap, with ISS and NISS showing notably higher AUPRC values, reflecting better performance in identifying true positives among the small proportion of deaths. In contrast, PHISE showed stronger relative performance for predicting red blood cell concentrate (RBCC) transfusion need, achieving an AUC of 0.77, closely approximating the values for ISS (0.83) and NISS (0.81; Fig. 3I) and more comparable areas under the precision-recall curve (Fig. 3J).

These results suggest that while PHISE does not fully match the performance of imaging-based scores like ISS and NISS, it retains meaningful predictive capacity for key clinical endpoints, especially in resource-limited or time-critical prehospital environments where imaging is unavailable.

### External validation in the TraumaRegister DGU® dataset

To assess the generalizability of PHISE, we externally validated the score in 14,136 trauma patients of the TraumaRegister DGU® from centers in Germany, Switzerland and Austria. This multinational registry includes prehospital severity assessments based on a simplified injury scoring system comparable to PHISE, although it distinguishes external soft tissue injuries as a separate anatomical region. The system aligns well with standard emergency physician documentation practices for injury severity in prehospital settings.

**Fig. 4:**
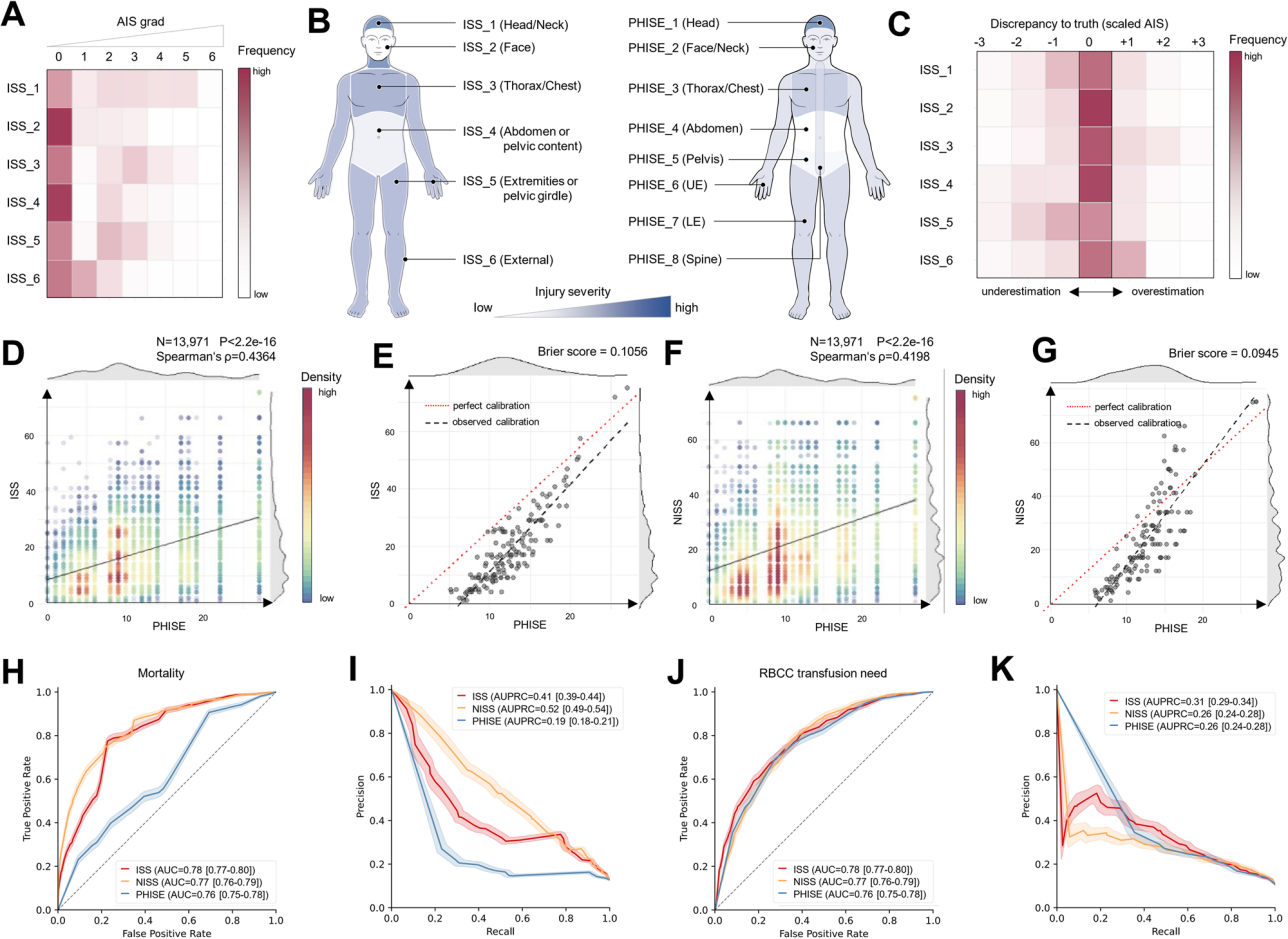
External validation of PHISE in the TraumaRegister DGU®. **A** Heatmap showing the distribution of injuries across ISS-defined body regions in the TraumaRegister DGU®. Cell color indicates frequency, stratified by AIS severity grade. **B** Schematic visualization of body regions affected by trauma, highlighting frequencies per region. Left: ISS-defined body regions. Right: PHISE-defined body regions. Darker colors represent higher injury frequencies. **C** Region-wise discrepancy between rescaled AIS severities and mapped PHISE grades across ISS body regions, replicating the comparison logic used in the derivation cohort. **D–G** Correlation and calibration of PHISE scores against traditional scores. Scatter plots illustrate the correlation of PHISE with ISS (D) and NISS (F). Panels E and G depict corresponding calibration curves using 100-patient bins; dashed lines indicate perfect calibration. **H**, **I** Receiver operating characteristics and precision-recall analyses for ISS, NISS, and PHISE in predicting in-hospital mortality. **J**, **K** Receiver operating characteristics and precision-recall analyses for ISS, NISS, and PHISE in predicting red blood cell concentrate (RBCC) transfusion need. Shaded areas represent 95% confidence intervals.

As in the NTDB® derivation cohort, we first conducted an exploratory analysis of injury distribution by body region and corresponding AIS severity levels (Fig. 4A). Anatomical overlays confirmed comparable injury frequencies and distribution patterns across ISS and PHISE region definitions (Fig. 4B). Region-wise discrepancy between rescaled AIS severities and PHISE again highlighted mismatches in the abdominal region. Interestingly, PHISE tended to overestimate severity in ISS region 6 (external), while underestimating injuries in ISS region 5 (extremities and pelvic girdle; Fig. 4C). Correlations between PHISE and traditional severity scores remained moderate (PHISE vs. ISS: Spearman’s ρ = 0.44; vs. NISS: ρ = 0.42; Fig. 4D, F). Calibration plots mirrored the trends observed in the derivation cohort, with PHISE tending to overestimate low-severity injuries and modestly underestimate the most severe cases (Fig. 4E, G). In outcome prediction, PHISE underperformed compared to ISS and NISS for in-hospital mortality (AUC: PHISE = 0.63, ISS = 0.81, NISS = 0.84 and AUPRC: PHISE = 0.19, ISS = 0.41, NISS = 0.52; Fig. 4H, I). However, it achieved near-to similar performance for predicting red blood cell concentrate (RBCC) transfusion need (AUC: PHISE = 0.76, ISS = 0.78, NISS = 0.77; AUPRC: PHISE = 0.26, ISS = 0.31, NISS = 0.26; Fig. 4J, K).

### Performance in Machine Learning models

We hypothesized that integrating the simplified PHISE score with additional prehospital variables into a machine learning (ML) model could mitigate the performance differences observed between PHISE and the traditional imaging-based severity scores. To test this, we embedded PHISE, ISS, and NISS separately into XGBoost-based gradient boosting models to predict two clinically relevant outcomes: in-hospital mortality and the need for red blood cell concentrate (RBCC) transfusion.

**Fig. 5:**
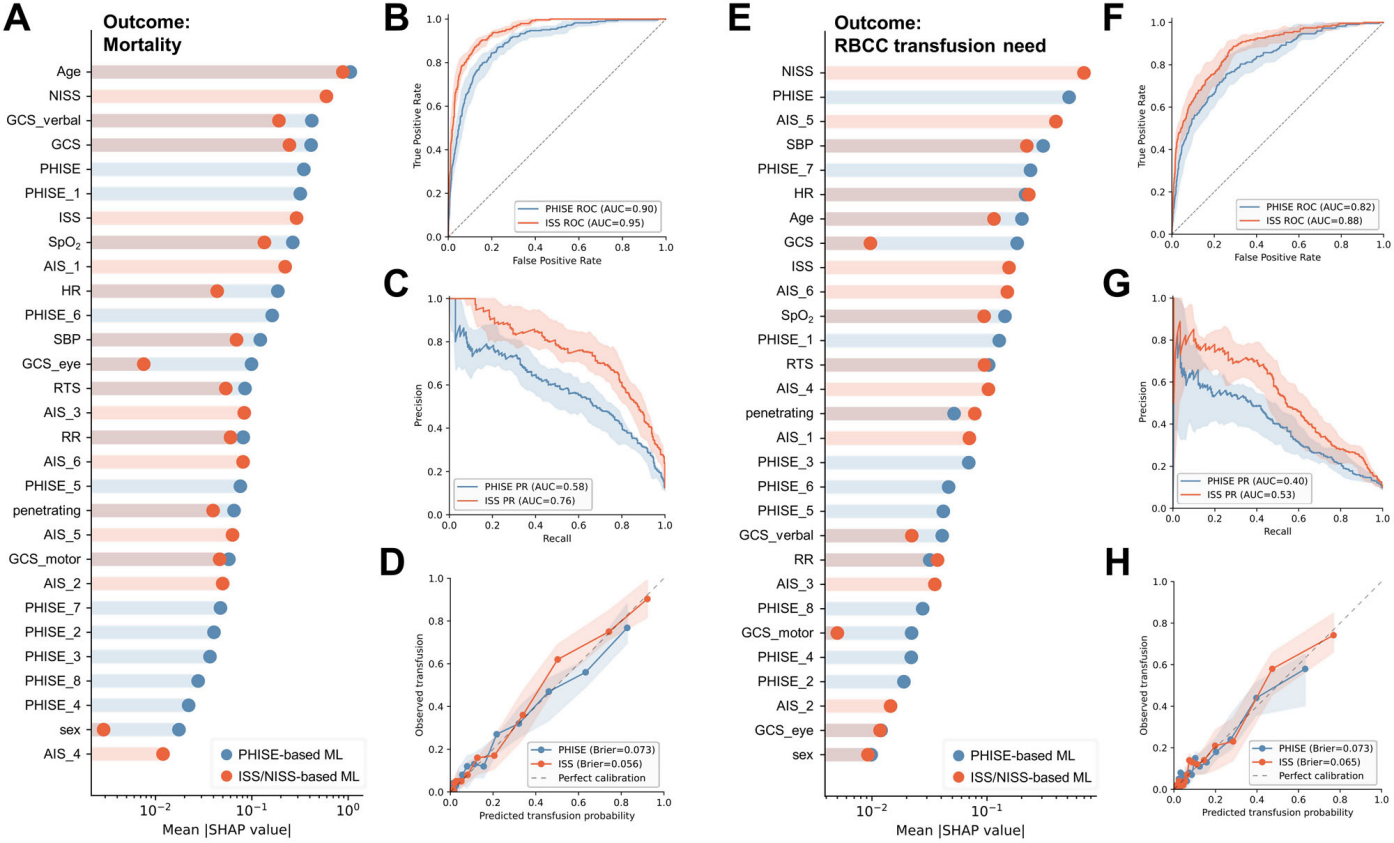
Integration of PHISE vs. ISS/NISS into machine learning models for mortality and transfusion prediction. **A**–**D** Performance comparison of XGBoost models predicting in-hospital mortality using either PHISE or traditional ISS and NISS as injury severity input. **A** SHAP summary plot ranking the most important predictors; both scoring systems and corresponding feature importance is shown. **B** ROC curve with area under the curve (AUC) values for PHISE- and ISS-based models. **C** Precision-recall (PR) curve with corresponding AUCs. **D** Calibration plot comparing predicted vs. observed mortality risk and Brier scores. **E**–**H** XGBoost model performance for prediction of red blood cell concentrate (RBCC) transfusion need. **E** SHAP summary plot showing feature importance for transfusion prediction. **F** ROC curve. **G** PR curve. **H** Calibration plot with predicted vs. observed probabilities and Brier scores. Shaded areas represent 95% confidence intervals.

For mortality prediction, performance differences between the three scores narrowed substantially in the ML context. However, minor differences in precision-recall performance (Fig. 5C) and calibration (Fig. 5D) remained, with ISS and NISS retaining a slight advantage. Notably, feature importance analysis revealed that the PHISE-based model extracted more value from the additional prehospital features compared to models using ISS or NISS, as suggestive by overall higher feature importances for the PHISE-based model (Fig. 5A). All shared variables demonstrated higher relative importance in the PHISE model, suggesting greater complementarity and model synergy. An exception was observed for the variable “penetrating injury mechanism,” which held similar or slightly higher importance in the ISS/NISS models. This trend was consistent in models predicting RBCC transfusion need. Again, PHISE-based models demonstrated similar predictive performance to ISS/NISS-based models, with negligible differences in AUC and calibration (Fig. 5F, G). Feature importance profiles were largely similar to those observed in the mortality models (Fig. 5E), further supporting the robustness of PHISE in integrated ML contexts. Detailed performance metrics can be found in Supplementary Table 1 and 2.

Additionally, we compared performance of the PHISE-based ML model to that of established scores for outcome prediction (Supplementary Fig. S5). The Revised Injury Severity Classification, version II (RISC2) developed to predict in-hospital mortality based on a combination of pre- and in-hospital variables, including the worst and second-worst AIS severities, head injury presence, age, sex, pupil reactivity and size, pre-injury health status, SBP, base excess, INR, hemoglobin level, and need for cardiopulmonary resuscitation^7,8^. The Trauma-Associated Severe Hemorrhage (TASH) score was designed to estimate the likelihood of massive transfusion and incorporates both prehospital and in-hospital variables such as systolic blood pressure, hemoglobin levels, intra-abdominal fluid, complex bone or pelvic fractures, heart rate, base excess, and sex^9^. Two additional established scores used for general outcome prediction are the Trauma Score and Injury Severity Score (TRISS)^10^ and reverse shock index multiplied by GCS (rSIG)^11^.

For mortality prediction (Supplementary Fig. S5A, B), RISC2 achieved the highest performance (AUC = 0.95), same as the PHISE-based ML model using ISS as the injury severity input. For RBCC transfusion prediction (Supplementary Fig. S5C, D), TASH yielded the best performance (AUC = 0.88), again same as the ISS-based ML model. In both scenarios, the PHISE-based models – despite relying only on prehospitally available variables—outperformed rSIG and TRISS, and achieved competitive results relative to scores that rely on in-hospital diagnostics.

Taken together, these findings support PHISE as a complementary, imaging-free anatomical input for early trauma stratification. When combined with prehospital variables such as vital signs, injury mechanism, and age into AI models, PHISE enables earlier outcome prediction with limited information loss compared with ISS/NISS-based models.

The ability to generate reliable predictions without waiting for lab results or imaging may offer critical time advantages in the early management of trauma patients at high risk of mortality or transfusion need.

## Discussion

This study introduces and validates the Prehospital Injury Severity Estimate (PHISE), a simplified trauma severity score designed for use without diagnostic imaging. Unlike the ISS and NISS, which are reliant on CT or other advanced imaging modalities, PHISE enables rapid scene-based assessment through clinical inspection and scene knowledge, filling a longstanding gap in trauma care where early decisions must be made before imaging results are available. PHISE should not be interpreted as a comprehensive patient-centered risk score. Like ISS and NISS, it primarily captures anatomical injury burden and therefore cannot fully account for physiological response, frailty, comorbidity, or the disproportionate impact of severe isolated injuries such as traumatic brain or spinal cord injury. Its intended role is to provide the missing anatomical injury estimate in the prehospital phase, which can then be combined with physiology, age, mechanism, and other clinical variables in AI-assisted decision models.

In both derivation (NTDB®) and validation (TR-DGU®) cohorts, PHISE demonstrated strong structural concordance with ISS and NISS. Robust calibration suggests that PHISE is capable of capturing injury burden with surprising fidelity given its reduced complexity. Importantly, the strongest agreement was found in regions accessible to clinical examination, such as the extremities and superficial injuries in general—further validating its feasibility for prehospital application.

While PHISE slightly underperformed ISS/NISS in predicting mortality, particularly in patients with severe internal injuries, it matched their performance in transfusion prediction—a clinically critical and time-sensitive outcome. Mortality was used mainly as a robust benchmark available in both registries; clinically actionable outputs include transfusion need, hemorrhage risk, trauma team activation, destination decisions, and early resource planning.

Most notably, when embedded into modern machine learning pipelines, PHISE performed nearly as well as ISS and NISS in both mortality and transfusion prediction. The addition of prehospital variables – vital signs, injury mechanism, and regional injury mapping – diminished the performance gap, underscoring how multimodal, AI-enabled models can compensate for PHISE’s lack of imaging data. In other words, once contextualized with real-time physiological and clinical features, the utility of traditional imaging-based scores diminishes, supporting the shift toward pragmatic, data-efficient categorization strategies.

Additionally, we benchmarked PHISE against established trauma scoring systems including RISC2, TASH and TRISS. These scores are well-established for outcome prediction but rely on in-hospital diagnostics such as lab results and imaging. PHISE-based ML models, which relied solely on prehospital data, outperformed TRISS and achieved comparable results to the scores incorporating blood testing and imaging. This supports the notion that PHISE enables early, imaging-independent prognostication at a level close to gold-standard models—but with the critical advantage of real-time availability in the prehospital phase. The potential time advantage of bypassing blood testing or imaging may directly benefit patients at high risk of bleeding or deterioration. Compared with simple physiological scores, PHISE adds structured regional anatomical information before imaging or laboratory results are available.

This finding is particularly relevant for emergency and critical care settings, where rapid decision-making and resource prioritization must occur before imaging is available. By providing a robust, structured, and AI-compatible injury severity estimate, PHISE holds promise as a frontline categorization tool. It offers opportunities for AI-enhanced dispatch, early transfusion protocols, direct-to-OR routing, and ICU resource planning – long before patients reach the CT scanner. Our findings also align with earlier efforts to simplify AIS-based injury scaling for more immediate clinical use: Civil and Schwab^3^ previously showed that condensed AIS charts could support accurate injury scaling in more than 95% of patients presenting to a Level I trauma center and might assist ISS calculation soon after admission. While this represented an important step toward bedside usability, it remained an in-hospital approach requiring injury identification after admission. PHISE extends this trajectory by shifting structured injury severity estimation further upstream into the prehospital setting, before diagnostic imaging is available. In a recent study, Choi et al. ^12^ attempted to address the problem of missing availability of the ISS by simplifying injury scoring based on typical injuries associated with each body region. These injury patterns were embedded into a multitask deep learning model to predict hospital length of stay, discharge disposition, and inpatient mortality. However, many of the injuries used in their model still require diagnostic imaging for confirmation. Thus, while their approach may enhance computational efficiency, it remains limited in real-world prehospital applicability – where immediate, imaging-independent assessment is essential for early triage and decision-making.

There are, however, limitations to consider.

PHISE relies on clinical examination and scene assessment, and its accuracy therefore depends on examiner experience and the quality of prehospital documentation. Subtle or occult injuries may still be missed, consistent with known limits of prehospital injury recognition^13^. Operationality, interrater reliability, time burden, workflow integration, and clinical impact were not assessed and require prospective EMS validation before routine implementation^14^.

Still, this work addresses a critical blind spot in trauma informatics: the absence of a validated, structured prehospital injury severity score. PHISE bridges that gap – offering both a practical standalone tool and a modular input for next-generation AI triage models.

## Methods

### Study design and setting

This retrospective, registry-based study was conducted using two large trauma datasets. The derivation cohort was sourced from the 2019 version of the Participant Use File (PUF, in .csv file format) of the National Trauma Data Bank® (NTDB®), representing trauma admissions from over 700 participating US hospitals. The NTDB® is a voluntary, hospital-reported registry that predominantly includes Level I and II trauma centers and may therefore overrepresent higher-resourced institutions. Only patients primarily admitted to the hospital were included in the study. Reported data include prehospital injury data alongside detailed in-hospital documentation on interventional or operative procedures or transfusions. The NTDB® captures a diverse trauma population, spanning all age groups and injury severities, with representation from urban and rural trauma centers. Transfusion of packed red blood cells within 4 h was considered as positive ground truth. To evaluate temporal stability and generalizability under atypical system conditions, we additionally analyzed the 2020 NTDB® dataset, which reflects trauma care during the COVID-19 pandemic. This secondary analysis (Supplementary Figs. S2–4) served as a stress test to assess robustness of PHISE performance despite potential shifts in trauma epidemiology and system-level resource constraints. Injury coding in the NTDB® cohort was based predominantly on AIS 2005, but also AIS 2015 codes. Review of the NTDB® AIS lookup files showed severity differences between coding variants in 45 injury codes (2.4%), affecting 3.3% of all patients; therefore, a relevant influence of AIS-version differences on the main analyses was considered unlikely.

For external validation, we used the TraumaRegister DGU® (TR-DGU®), a multinational European trauma registry operated by the German Trauma Society (DGU). The TraumaRegister DGU® sample consisted of data from Germany, Switzerland and Austria. The register records all cases of critically injured pediatric and adult patients with prehospital vital signs that were admitted to the hospital via the emergency trauma room. Indications for trauma room comprise severe ABCDE problems, unstable, penetrating or severe injuries including burns as well as specific trauma mechanisms (fall from ≥3 meters, ejection from car), according to the German guidelines for treatment of severely injured patients^15^. Non-primary patients were excluded from the study. Extracts from six German (Tübingen, Freiburg, Stuttgart, Essen, Duisburg, Ludwigshafen), one Austrian (Graz) and one Swiss (Zürich) university trauma centers were pooled from previously anonymized datasets in accordance to local data protection rules and ethical responsibility (project number 569/2023 A). The register contains detailed information on demographics, injury patterns, comorbidities, pre-hospital and clinical management, intensive care unit course and important laboratory findings including transfusion data. Transfusion of blood products in the emergency department or the operating room were considered as positive ground truth.

The study follows TRIPOD + AI guidelines for transparent reporting and validation of diagnostic and prognostic models involving artificial intelligence, ensuring methodological rigor and reproducibility. The study was conducted in accordance with the Declaration of Helsinki and relevant local data protection regulations. TraumaRegister DGU® data were obtained from previously anonymized extracts across participating centers. The use of both the NTDB® and TraumaRegister DGU® datasets was approved in accordance with institutional ethical oversight and data governance policies (Ethics Committee at the Medical Faculty of Eberhard Karls University and at Tübingen University Hospital, project number 569/2023 A). The analyzed registry datasets were fully anonymized and de-identified prior to access by the investigators. Therefore, the requirement for individual informed consent was waived by the ethics committee due to the retrospective nature of the study and the exclusive use of anonymized registry data.

### Population and inclusion criteria

Patients were included if they had complete anatomical injury coding based on the Abbreviated Injury Scale (AIS) and available core demographic and prehospital data. In NTDB®, we included patients with AIS-defined injuries and a complete ISS/NISS calculation. NISS was calculated for each patient as the sum of the squares of the three highest AIS severity scores, irrespective of body region, following the original NISS definition^2^. This calculation was performed using the available AIS-coded injury data including severity levels.

In TraumaRegister DGU®, a simplified prehospital injury scoring system is routinely recorded by EMS teams, allowing for external validation of PHISE under real-world prehospital conditions. Patients with incomplete records or implausible values were excluded.

### PHISE score derivation

PHISE is a body region-based score designed to reflect injury severity based solely on clinical examination and scene/trauma mechanism knowledge. It assigns a severity grade (0: none, 1: minor, 2: moderate, 3: severe) on eight body regions (brain/skull, face/neck, thorax, abdomen, pelvis, spine, upper extremity and lower extremity).

To simulate scene-based scoring using retrospective AIS data, we developed a rule-based mapping system. Detailed AIS-coded injury descriptions were analyzed using natural language processing (NLP) and Large Language Model (LLM) inference (GPT-4) to (1) assign each AIS code to a PHISE body region, and (2) determine whether the injury could be identified by clinical gestalt or required imaging.

In detail, to accomplish task (1), rule-based NLP was used to assign body parts to corresponding PHISE body regions. Task (2) was done by a detailed prompt (Supplementary Fig. 1) to determine if imaging was required to diagnose the specific injury, or if the injury could be determined from clinical examination, gestalt, knowledge of trauma mechanisms and scene circumstances. For cases relying on imaging or undetermined cases, a random severity grade was given. Else, for assessable injuries, AIS severity (0–6) was mapped to PHISE grades (0–3) using predefined rules (Fig. 2C). Injuries requiring imaging were randomly assigned PHISE grades based on frequency distributions. Multiple injuries per patient were aggregated by summing the squares of the three highest PHISE regional scores, analogous to ISS logic.

### Reference scores: ISS and NISS

ISS and NISS were calculated using standard rules based on the AIS 2005 Update 2008 coding system. ISS is defined as the sum of squares of the highest AIS score in the three most severely injured ISS body regions. NISS instead sums the squares of the three most severe injuries regardless of region. ISS/NISS were AIS-based in both registries; however, NTDB® and TraumaRegister DGU® differ in registry structure, inclusion criteria, and coding workflow, so both datasets were analyzed separately.

### Outcomes

The primary outcome was in-hospital mortality, selected because it is robustly captured in both registries. Secondary outcomes included red blood cell concentrate (RBCC) transfusion need, hospital length of stay (LOS), and ICU LOS. In TR-DGU®, transfusion was defined as any administration of RBCs within the first 24 h of admission.

### Supervised machine learning

Supervised machine learning was carried out in Python and was implemented by scikit-learn (1.3.2), scikit-learn-extra (0.3.0) and scikit-misc (0.3.1). Multiple machine learning models were tested (Logistic Regression, Random Forest, Support Vector Machine, XGBoost, AdaBoost, light GBM, Naïve Bayes, Multilayer Perceptron), among which XGBoost showed best performance and suitability. Prior to ML model training, the derivation cohort was randomly split into a training (80% of cases) and validation (20% of cases). Missing values were then imputed by mean for continuous data, median for ordinal data and modus for categorical data. Features were scaled using scikit-learn’s ColumnTransformer. Using a nested cross-fold validation approach a classifier was trained including hyperparameter tuning and 10-fold cross-validation. Subsequently, the trained classifier was applied to previously unseen data of the internal validation dataset. Classification metrics were calculated using a 1000-fold bootstrap approach. Performance was evaluated by sensitivity, specificity, PPV, NPV, and F1-score. For better model interpretability, SHAP values (SHapley Additive exPlanations) were calculated for each blood product. The feature importance for each variable was scaled to lay between 0 and 1, the mean importance per feature was computed globally on the validation split and features were ranked in descending order.

### Statistical analysis

Correlation between PHISE and ISS/NISS was assessed using Spearman’s rank correlation coefficient. Calibration was evaluated using bin-wise average plots (1000-patient bins), comparing average PHISE and ISS/NISS scores per bin. ROC analyses were performed for binary outcomes (mortality, transfusion) using the pROC package, and differences in AUC were compared using DeLong’s test. Continuous outcomes were analyzed using Spearman correlation and linear modeling.

For multivariable prognostic analysis, we trained XGBoost classifiers using either ISS, NISS, or PHISE as severity input alongside other prehospital features (age, sex, GCS, injury mechanism, and vital signs). Models were trained on the NTDB® and tested on TR-DGU®. Hyperparameters were optimized using cross-validation, and calibration was visualized using reliability plots.

All analyses were performed in R (version 4.3.1) and Python (version 3.11) with significance set at *p* < 0.05.

## Supplementary information

Supplementary Information

## Data Availability

Data from the National Trauma Data Bank® can be purchased from https://www.facs.org/quality-programs/trauma/quality/national-trauma-data-bank/. Data extracts from the TraumaRegister DGU® may be requested from the corresponding author upon reasonable request and in case of approval from the DGU. Access to data requires application and positive ethical approval.

## Code availability

All analyses were performed in R (version 4.3.1) via RStudio Desktop (version 2026.06.0) and Python (version 3.11) via Jupyter Notebook (version 7.6.0). The R and Python code to reproduce the figures are made available upon request.
