# Supplementary material for: Prehospital Injury Severity Estimate (PHISE) matches in-hospital trauma scores when embedded in AI models
Source: NPJ Digit Med. 2026 Aug 7;9:607. doi: 10.1038/s41746-026-03074-7 (PMC13448184; doi:10.1038/s41746-026-03074-7)
Supplement: Supplementary file 1 [file 41746_2026_3074_MOESM1_ESM.pdf]

Supplemental Material to

Prehospital Injury Severity Estimate (PHISE) matches in-hospital trauma scores when embedded in AI models

Running title: Development and external validation of a simplified, scene-based injury severity score offering ISS-like performance without CT imaging requirements

Manuel Sigle MD PhD[1], Andreas Goldschmied MD[1], Prof Meinrad Gawaz MD[1], Alexander Münch MD[2], Prof Peter Rosenberger MD[2], preTransIT Investigator group*, Robert Wunderlich MD, MSc[2,#]

*preTransIT Investigator group:
Sven Weisser MD[3], Andreas Gather MD[4], Prof Alfred Grützner MD[4], Oliver Kamp MD[5], Prof Christian Waydhas MD[5], Prof Marcel Dudda MD[5,6], André Nohl MD[5,7], Nicolas Eibinger, MD[8], Prof Paul Puchwein MD[8], Saffet Özkaya MD[9], Prof Christian Knop MD[9], Martin Breitkopf MD[10], Kai Oliver Jensen MD[11], Yannik Kalbas MD[11], Ferdinand C Wagner MD[12], Benjamin Erdle MD[12]

[1]Department of Cardiology and Angiology, University Hospital Tübingen, Eberhard Karls University Tübingen, 72076, Tübingen, Germany
[2]University Department of Anesthesiology and Intensive Care Medicine, University Hospital Tübingen, Eberhard Karls University Tübingen, 72076, Tübingen, Germany
[3]Department for Paraplegics, technical orthopedics and spinal surgery, BG Tübingen Hospital, Tübingen, Germany.
[4]BG Ludwigshafen Hospital, Ludwigshafen, Germany
[5]Department of Trauma, Hand and Reconstructive Surgery, University Hospital, Essen, Germany
[6]Department of Orthopedics and Trauma Surgery, BG Klinikum Duisburg, University of Duisburg-Essen
[7]Zentrum für Notfallmedizin, BG Klinikum Duisburg, Duisburg, Germany.
[8]Department of Orthopedics and Trauma, Medical University of Graz, Graz, Austria.
[9]Department of Trauma Surgery and Orthopedics, Klinikum Stuttgart-Katharinenhospital, Stuttgart, Germany.
[10]Department of Anesthesiology, operative Intensive Care Medicine, Emergency Medicine and Pain Medicine, Klinikum Stuttgart-Katharinenhospital, Stuttgart, Germany.
[11]University Hospital Zürich, Trauma Department Zürich, Zürich, Switzerland.
[12]Department of Orthopedics and Traumatology, Freiburg University Hospital, Freiburg, Germany

# corresponding author
Robert Wunderlich, MD, EMDM
University Department of Anesthesiology and Intensive Care Medicine, University Hospital Tübingen, Hoppe-Seyler-Straße 3, 72076 Tübingen, Germany
Robert.Wunderlich@med.uni-tuebingen.de
+49 7071 29-86622

Table of Content

Content	Description	Page
**Supplemental Tables**	**S1:** Classification metrics for outcome mortality	3
	**S2:** Classification metrics for outcome RBCC transfusion need	4
**Supplemental Figures**	**S1**: Prompt for LLM-assisted evaluation of imaging need	5
	**S2:** Comparison of prevalence, severity and imaging need of top injuries in the NTDB® 2020 dataset.	6
	**S3:** PHISE correlation and calibration in the NTDB® 2020 dataset.	7
	**S4:** PHISE outcome prediction in the NTDB® 2020 dataset.	8
	**S5:** Predictive performance of established trauma scores for mortality and transfusion need based on the TraumaRegister DGU® dataset.	9

Supplemental Tables

Supplemental Table S1. Classification metrics for outcome mortality

		AUC (mean, 95% CI)	Accuracy (mean, 95% CI)	Precision (weighted) (mean, 95% CI)	Recall (weighted) (mean, 95% CI)	Specificity (mean, 95% CI)	NPV (mean, 95% CI)	F1 Score (weighted (mean, 95% CI)	PRC (mean, 95% CI)
PHISE	train	0.92 (0.91-0.92)	0.79 (0.78-0.8)	0.91 (0.91-0.91)	0.79 (0.78-0.8)	0.78 (0.77-0.78)	0.99 (0.98-0.99)	0.85 (0.84-0.85)	0.61 (0.58-0.65)
	test	0.89 (0.87-0.91)	0.77 (0.76-0.79)	0.9 (0.88-0.91)	0.77 (0.76-0.79)	0.76 (0.74-0.78)	0.97 (0.96-0.98)	0.83 (0.82-0.84)	0.57 (0.53-0.61)
ISS	train	0.94 (0.94-0.95)	0.86 (0.85-0.86)	0.92 (0.92-0.92)	0.86 (0.85-0.86)	0.85 (0.84-0.86)	0.98 (0.98-0.99)	0.89 (0.88-0.89)	0.72 (0.69-0.75)
	test	0.95 (0.94-0.96)	0.86 (0.85-0.87)	0.92 (0.91-0.92)	0.86 (0.85-0.87)	0.85 (0.84-0.86)	0.98 (0.98-0.99)	0.88 (0.88-0.89)	0.76 (0.73-0.8)

Supplemental Table S2. Classification metrics for outcome RBCC transfusion need

		AUC (mean, 95% CI)	Accuracy (mean, 95% CI)	Precision (weighted) (mean, 95% CI)	Recall (weighted) (mean, 95% CI)	Specificity (mean, 95% CI)	NPV (mean, 95% CI)	F1 Score (weighted (mean, 95% CI)	PRC (mean, 95% CI)
PHISE	train	0.83 (0.82-0.84)	0.79 (0.78-0.8)	0.89 (0.89-0.9)	0.79 (0.78-0.8)	0.8 (0.79-0.81)	0.96 (0.96-0.97)	0.84 (0.83-0.85)	0.39 (0.36-0.42)
	test	0.82 (0.79-0.85)	0.78 (0.77-0.8)	0.89 (0.87-0.9)	0.78 (0.77-0.8)	0.8 (0.78-0.81)	0.96 (0.95-0.97)	0.83 (0.82-0.85)	0.39 (0.32-0.46)
ISS	train	0.87 (0.86-0.88)	0.79 (0.78-0.8)	0.9 (0.9-0.91)	0.79 (0.78-0.8)	0.79 (0.78-0.8)	0.97 (0.97-0.97)	0.84 (0.84-0.85)	0.49 (0.46-0.52)
	test	0.88 (0.85-0.9)	0.8 (0.78-0.82)	0.9 (0.89-0.91)	0.8 (0.78-0.82)	0.8 (0.79-0.82)	0.97 (0.96-0.98)	0.85 (0.83-0.86)	0.53 (0.46-0.6)

Supplemental Figures

Figure S1

You are an experienced clinician in the field of traumatology. You get comma-separated data containing AIS (abbreviated injury scale) data with these columns:

- “AISPREDOT” (AIS code)
- “AISSEVERITY” (1=Minor, AIS 2=Moderate, AIS 3=Serious, AIS 4=Severe, AIS 5=Critical, AIS 6=Maximal (currently untreatable))
- “AISDESCRIPTION” (text description of the injury)
- “AISVERSION” (year of AIS definition)
- “ISSREGION” (1=Head, 2=Face, 3=Thorax, 4=Abdomen, 5=Extremities/Pelvis & Soft Tissue, 6=External)

The aim is to identify which injuries can be approximated prehospitally without imaging.
For each row, review “AISDESCRIPTION” and decide using your expert reasoning if a prehospital provider can approximate the injury without imaging (clinical exam or scene context), or if imaging is explicitly required (no reliable proxy in the field). Then:
1. Add a column named needForImaging
2. Assign one of:
   - 0 = No imaging needed (purely external/superficial), as "AISDESCRIPTION" can be approximated by clinical exam or mechanism/scene (e.g., obvious long-bone fracture); or imaging can be neglected for diagnosis at least in the prehospital setting
   - 1 = Imaging (CT, X-ray, angiography, etc.) is the only reliable way to confirm that exact "AISDESCRIPTION", no other reliable proxy exists
   - 2 = Neither 0 nor 1 clearly applies, individual evaluation needed. Use only when the injury description is ambiguous and does not match any clear rule for 0 or 1.

Do not assign 1 if it is generally recommended to perform imaging: Only assign 1 if imaging is required to make the diagnosis as described in "AISDESCRIPTION" and it is not possible to do so by clinical exam, visual inspection and knowledge about the scene context.

General rules:

- “NFS” means Not Further Specified. When it’s purely external or superficial (e.g., “Injuries to the Head NFS” with no focal signs), you may code 0 if no exam or a basic exam/mechanism assessment suffices to make the diagnosis
- Based on the AIS severity you can assess the impact on internal organs (e.g., "Whole body injury (explosion-type) NFS" can have a severity of 2,4, or 6, where 6 is most probable to cause additional internal injury which would require imaging for diagnosis).
- For suspected fractures (extremities, long bone), assume palpation/inspection is diagnostic → 0, unless truly occult → 1. Pelvis fractures are often occult → 1.
- Only code 1 if you cannot reliably make the diagnosis in the field. Examples:
  - Cerebral artery thrombosis/occlusion/aneurysm
  - Cerebral venous sinus fistula/thrombosis/occlusion
  - Injury to any internal thoracic or abdominal organ (lung, heart, spleen, liver, kidney, bowel, retroperitoneum) with nonspecific signs or obvious penetration
  - Injury to veins or arteries in the abdomen/pelvis; this is also relevant for veins/arteries NFS
  - Spine/vertebral injury without any neurologic deficit
- Small hand/foot fractures, or joint dislocations → 0.
- Fractures/injuries involving superficial structures or bones that can be touched → 0.
- Penetrating injuries which can be clearly seen → 0.
- Clinical states (e.g. awake, unconscious) → 0.

Your task is to add the column "needForImaging" for every row. Please use your medical knowledge and expert reasoning as an AI, do not create a Python script that filters based on common patterns.

Figure S1: Prompt for LLM-assisted evaluation of imaging need

ChatGPT model GPT-4o-mini (April 2025) was used for this prompt.

Figure S2

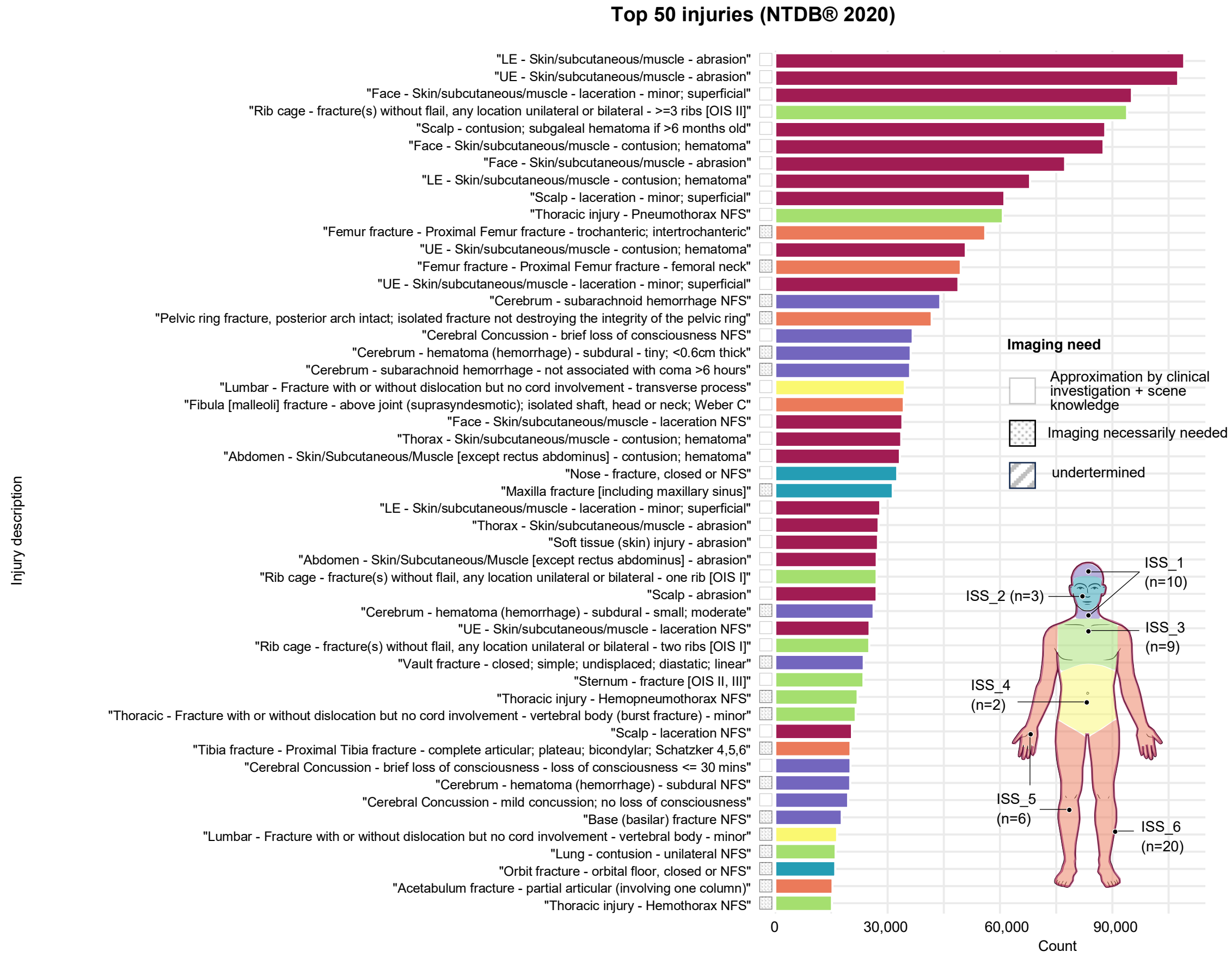

Figure S2: Comparison of prevalence, severity and imaging need of top injuries in the NTDB® 2020 dataset.

Distribution of the 50 most frequent injury codes in the NTDB® 2020 dataset, categorized by body region (bar color). An anatomical reference map displays the mapping of AIS-defined body regions per color to the six ISS body regions. The texture indicator refers to the estimated imaging need, as identified using a Large Language Model.

Figure S3

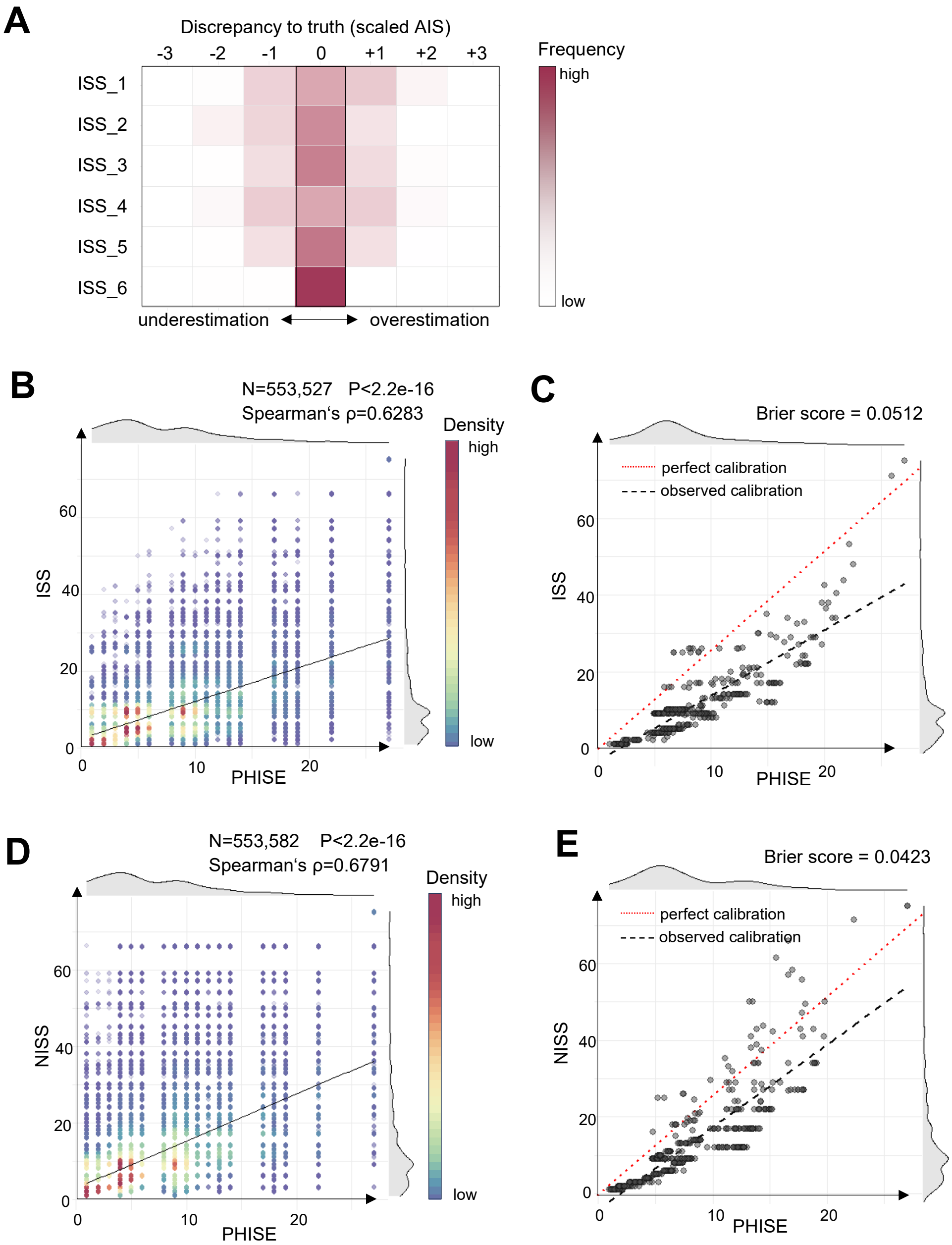

Figure S3: PHISE correlation and calibration in the NTDB® 2020 dataset.

A Region-wise discrepancy between rescaled AIS severities and mapped PHISE grades across all ISS body regions. ISS region 6 (external injuries) showed no discrepancy, as all relevant injuries can be identified without imaging. **B-E** Correlation and calibration of PHISE scores against traditional scores. Scatter plots show correlation of PHISE with ISS (B) and NISS (D). Panels C and E depict corresponding calibration plots using 1,000-patient bins; the dashed line indicates ideal calibration.

Figure S4

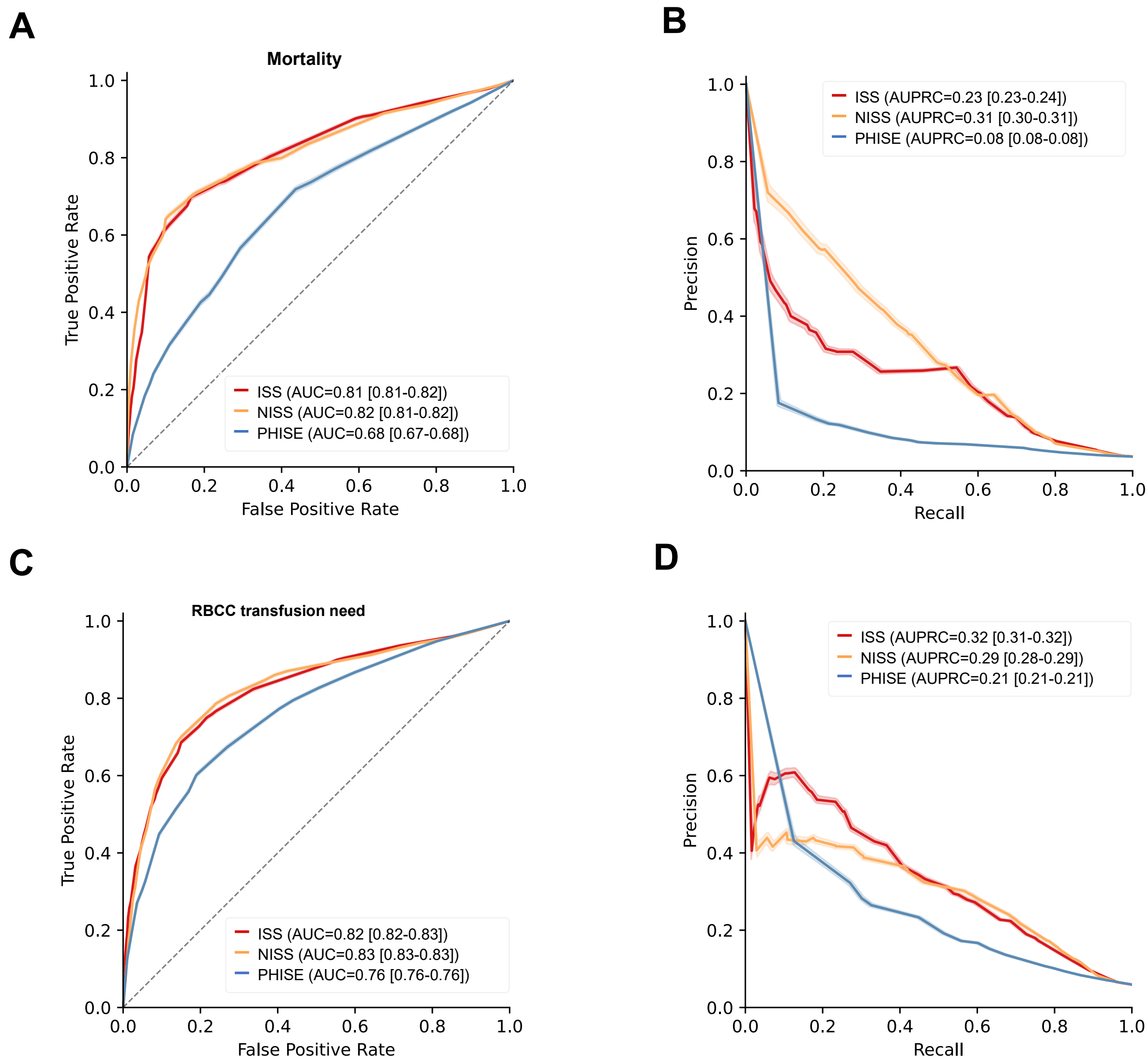

Figure S4: PHISE outcome prediction in the NTDB® 2020 dataset.

A, B Receiver operating characteristics and precision-recall analyses for ISS, NISS, and PHISE in predicting in-hospital mortality. **C, D** Receiver operating characteristics and precision-recall analyses for ISS, NISS, and PHISE in predicting red blood cell concentrate (RBCC) transfusion need. Shaded areas represent 95% confidence intervals.

Figure S5

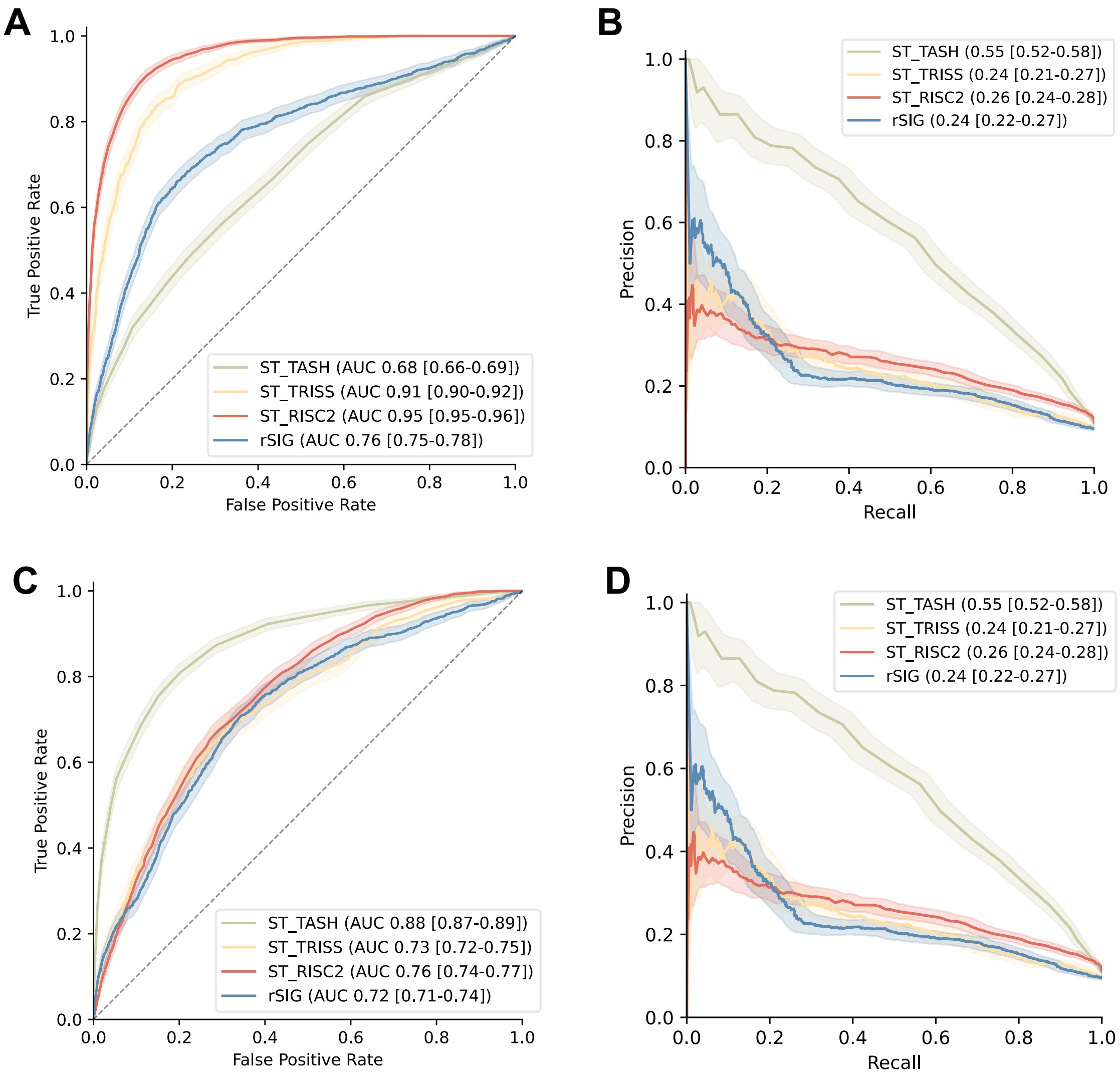

Figure S5. Predictive performance of established trauma scores for mortality and transfusion need based on the TraumaRegister DGU® dataset.

A, B Receiver operating characteristics and precision-recall analyses for in-hospital mortality prediction.

C, D Receiver operating characteristics and precision-recall analyses for red blood cell concentrate transfusion need.

Shaded areas represent 95% confidence intervals. RISC2 – Revised Injury Severity Classification, version II; TASH – Trauma-associated severe hemorrhage; TRISS – Trauma Score and Injury Severity Score; rSIG – reverse shock index multipled by GCS
